# Etiologies and Outcomes of Acute Respiratory Failure in Patients Treated with Immune Checkpoint Inhibitors

**DOI:** 10.21203/rs.3.rs-9096889/v1

**Published:** 2026-04-24

**Authors:** Rupali Sood, Kevin J. Psoter, Aliyah Pabani, Cheng Ting Lin, Mohammed Ghanbar, David N. Hager, Sonye Danoff, Karthik Suresh, Chad H. Hochberg

**Affiliations:** The Johns Hopkins Hospital

**Keywords:** immune checkpoint inhibitors, respiratory failure, checkpoint inhibitor pneumonitis

## Abstract

**Background:**

Indications for immune checkpoint inhibitors (ICIs) are expanding across cancer types and stages. While this carries promise for improved survival, increasing use has raised concern for respiratory complications in patients with multi-morbidities. Pulmonary complications such as checkpoint inhibitor pneumonitis (CIP), a recognized immune-related ICI toxicity, can lead to severe acute respiratory failure (ARF). However, not all ARF following ICI exposure is attributable to CIP, and while different causes imply distinct management and prognostic implications, the types of complications leading to ARF after ICI remain poorly understood.

**Methods:**

We conducted a retrospective cohort study of ICI-treated adults who developed ARF, defined as ≥ 24 hours of invasive or noninvasive ventilatory support within 2 years of first ICI receipt, treated at five hospitals in a single health system (2017–2024). ARF etiology was determined by manual chart review, and suspected CIP cases underwent multidisciplinary adjudication. We evaluated associations between CIP versus non-CIP ARF and 90-day all-cause mortality using multivariable Cox proportional hazards models.

**Results:**

Among 197 ICI-treated patients with ARF, the median age was 66 years, and the most frequent cancer type was non-small cell lung cancer. Among these patients, 29 (15%) had CIP, and 168 (85%) had other etiologies of ARF (e.g., pneumonia, progressive cancer, aspiration, fluid overload). In the first 24 hours, CIP patients exhibited more severe hypoxemia but less multiorgan dysfunction than non-CIP patients. Ninety-day mortality was 66% for CIP and 71% for non-CIP ARF. In adjusted analyses, CIP vs. non-CIP ARF was not significantly associated with 90-day mortality (adjusted hazard ratio [aHR] = 0.66; 95% CI: 0.40–1.08) but was associated with decreased in-hospital mortality (aHR = 0.43; 95% CI: 0.21–0.88). Among ARF survivors, 13% restarted ICI therapy: 5% of adjudicated CIP vs 17% of adjudicated non-CIP (p = 0.28); only 7% of suspected but non-adjudicated CIP cases restarted ICI.

**Conclusions:**

In this cohort of ICI-treated patients who developed acute respiratory failure, most ARF was not attributable to CIP. While CIP and non-CIP ARF had similar 90-day mortality, CIP-related respiratory failure was associated with lower in-hospital mortality and lower likelihood of ICI continuation, underscoring the importance of accurate etiologic attribution in this vulnerable group.

## BACKGROUND

Immune checkpoint inhibitors (ICIs) are a class of cancer therapeutics that can generate durable responses in difficult-to-treat malignancies. In 2017, approximately 44% of patients with cancer in the United States were eligible for ICI therapy, and indications have continued to expand since that time and across a spectrum of strategies from curative to palliative intent.^1-3^ As ICIs are increasingly used across cancer types and in patients with multiple comorbidities, understanding the presentation and targeted management of treatment-related toxicities is an important clinical consideration.^4-8^

While pulmonary complications of ICI-therapy have been described, there is little known about which complications yield severe acute respiratory failure (ARF), which is generally likely to be life-threatening, and may have significant implications for survivors in regards to ongoing cancer care.^7^ In oncologic patients broadly, ARF has many causes, including infections, pulmonary embolism, exacerbation of underlying lung disease, and progressive malignancy. In ICI-treated patients, ICI-related specific pulmonary toxicities include inflammatory lung injury, termed checkpoint inhibitor pneumonitis (CIP), in addition to other inflammatory-related sequelae such as sarcoid-like granulomatous disease, and serositis-related pleural effusions. In addition, non-pulmonary immune-related adverse events, including myocarditis and neuromuscular toxicity may precipitate ARF indirectly and further complicate diagnostic and management decisions in ICI treated patients presenting with ARF.^9-19^ While, these etiologies of ARF differ substantially in diagnostic approach, management strategies, and implications for continuation of ICI therapy,^20^ the etiologic distribution, and outcomes of severe ARF following ICI exposure remain incompletely characterized.

Limited data exist to inform whether outcomes differ when ARF is attributable to CIP versus alternative etiologies, particularly among patients with severe disease requiring ventilatory support.^14, 20-22^ To address this gap, we conducted a multihospital retrospective cohort study of ICI-treated patients with severe ARF to describe ARF etiologies and to evaluate the association between CIP versus non-CIP ARF and survival. We hypothesized that CIP-related ARF is associated with better survival compared to non-CIP-related ARF.

## METHODS

### Study Design and Cohort

We conducted a retrospective cohort study across two academic and three community hospitals within the Johns Hopkins Health System (JHHS). Data were extracted from an electronic health-record (EHR) registry of patients ever admitted to intensive care unit or intermediate care units in the JHHS and was housed on the Johns Hopkins Precision Medicine Analytics Platform (PMAP).^23^ We included adults (≥ 18 years old) treated with ICIs in an inpatient or outpatient JHHS setting between July 1, 2017, and December 31, 2024, and who developed ARF within 2 years after first ICI exposure. This time window for ARF was selected based on prior reports of the range for CIP-related respiratory failure.^24^ We pragmatically defined ARF as receipt of advanced respiratory support, either invasive mechanical ventilation (IMV) or non-invasive ventilation (NIV), including non-invasive positive-pressure ventilation (NIPPV) or high-flow nasal oxygen (HFNO), for ≥ 24 continuous hours. This threshold excludes brief procedural use and selects for clinically significant ARF as signified by persistent respiratory support requirements. For NIV, breaks of ≤ 2 hours off NIV support were allowed to capture patients who are intermittently but persistently supported with NIV over a 24-hour period. All patients meeting initial inclusion criteria were manually chart reviewed by author R.S. (pulmonary and critical care physician), and patients supported with IMV for non-respiratory failure related reasons (e.g. post-procedural, altered mental status only) were excluded.

The cause of ARF was adjudicated by manual chart review of clinical documentation, laboratory results, and imaging (author R.S). Although multiple etiologies could coexist, patients were classified into mutually exclusive groups according to the clinically-suspected primary clinical driver: aspiration, suspected CIP, neuromuscular weakness, pneumonia (defined as positive respiratory viral panel, and/or positive bacterial or fungal respiratory culture), progressive cancer, pulmonary embolism, sepsis-related respiratory failure (defined as positive non-respiratory culture with other signs of sepsis), or fluid overload.

CIP was diagnosed in accordance with national and international guidelines, including using a best practice of multi-disciplinary adjudication.^17, 25-27^ Patients were initially classified as having suspected CIP if the treating clinical team documented concern for immune checkpoint inhibitor-associated pneumonitis and initiated management for CIP (e.g. corticosteroid therapy and/or discontinuation of immune checkpoint inhibitor therapy). For each suspected CIP patient, we retrospectively determined CIP diagnosis via multidisciplinary adjudication by a panel including two board-certified pulmonologists, one oncologist, and one thoracic radiologist. A CIP diagnosis required compatible clinical features (new or worsening dyspnea, cough, or hypoxemia), characteristic radiologic patterns (ground-glass opacities, patchy consolidations, or organizing-pneumonia pattern), and exclusion of alternative etiologies through targeted microbiologic testing, including bronchoalveolar lavage studies when available, and comprehensive assessment for alternative etiologies including tumor progression, or other treatment-related lung injury (**Appendix A**).^17, 25-27^ We evaluated inter-rater reliability among multidisciplinary reviewers using Cohen’s κ statistic.

### Covariates

We extracted covariates electronically from the EHR registry and via manual chart review. Extracted variables included demographic characteristics (age, sex, race, ethnicity, body mass index [BMI], smoking history, hospital type [academic/community]), and comorbidities (Elixhauser comorbidity count). Cancer-specific covariates included cancer type, stage, and presence of brain metastases. ICI-specific data included which ICI was administered, cumulative instances of any ICI administration, and timing of administrations. We also assessed for receipt of concurrent chemotherapy, thoracic radiation (palliative or definitive intent), or tyrosine kinase inhibitors (TKIs), as these have been linked to CIP in prior studies.^28, 29^ We included the following acute-illness variables: type of respiratory support (NIV or IMV), presence of viral infection, lowest oxygen saturation-to-fraction-of-inspired-oxygen ratio (SpO_2_/F_I_O_2_ [S/F]) within the first 24 hours of ARF, non-respiratory Sequential Organ Failure Assessment (SOFA) score, vasopressor use, and presence of other non-pulmonary immune related adverse event (irAE) during admission. We additionally captured data related to other therapies received during hospitalization including administration of intravenous antibiotics for ≥ 2 days after start of ARF, corticosteroid use and dosing (≥ 1 mg/kg methylprednisolone equivalent, which is the recommended dosing for treating CIP^30^), and use of additional immunosuppressive therapies including intravenous immunoglobulin (IVIG), infliximab, tocilizumab, and mycophenolate mofetil. For SOFA sub-scores (central nervous system, cardiovascular, liver, coagulation, and renal), we imputed missing values as normal, otherwise no missing data were imputed, and we performed complete-case analyses throughout (**Table E1**).

### Exposures and Outcomes

The primary exposure was ARF from CIP versus non-CIP causes. The primary outcome was 90-day all-cause mortality following the onset of ARF. Vital status for patients discharged from the hospital alive before day 90 was determined by linkage to the national death records.^31^ Secondary outcomes included in-hospital mortality, and time to hospital discharge accounting for competing risk of death. For time-to-event analyses, individuals entered risk sets at ARF onset and were followed until death or administrative censoring at 90 days for the primary outcome, until in-hospital death or discharge for in-hospital mortality, and until hospital discharge for length of stay, with death treated as a competing event using Fine-Gray sub-distribution hazard models.^32^

### Statistical Analysis

We summarized and compared baseline characteristics and clinical outcomes between patients diagnosed with CIP vs non-CIP ARF using Wilcoxon rank sum, and χ^2^ or Fisher exact tests for continuous and categorical variables, respectively. Time to death and discharge for the CIP and non-CIP ARF groups were plotted using Kaplan-Meier curves and compared using a log-rank test.

We used unadjusted and multivariable Cox proportional-hazards regression to evaluate the association between CIP status and 90-day mortality as well as in-hospital mortality. Covariates for adjusted models were pre-specified based on prior literature and expert study team consensus and were guided by a directed acyclic graph (**Figure E1**). These included age, sex, Elixhauser comorbidity count, smoking status, presence of other immune-related adverse events, cancer type (lung vs. other), and cancer stage. The proportional hazards assumption was verified for all models. Results are presented as hazard ratios (HRs) with 95% confidence intervals (CIs), where a HR < 1.0 indicates a lower hazard of death (better survival). For the secondary outcome of time-to-hospital discharge alive, in-hospital death was treated as competing risk using a Fine-Gray sub-distribution model.^32^ Competing risk model estimates are presented as sub-distribution hazard ratios (sHRs), in which a sHR > 1.0 indicates faster time to discharge alive.

As exploratory analyses, we evaluated patterns of ICI rechallenge among patients who survived to hospital discharge after the initial ARF episode. We stratified these analyses by CIP versus non-CIP adjudicated diagnosis, and by treatment for suspected CIP versus not during hospitalization. We also conducted several sensitivity analyses. These included analyses adding additional covariates for prior cancer-directed therapies to assess for potential treatment-related confounding, and included: concurrent chemotherapy with the ICI, definitive thoracic radiation (defined as curative-intent radiation to the thorax) prior to ARF event, TKI exposure prior to ARF, and ICI exposure duration (number of days from first to last ICI dose prior to ARF). We also repeated the primary analysis in a subset of patients with non-small cell lung cancer (NSCLC), the most common underlying malignancy in this cohort, to evaluate whether findings were consistent within this more homogeneous cancer population. Lastly, we evaluated the primary and secondary outcomes in models stratified by oxygenation status using a cutoff of SpO_2_/FiO_2_ ≤ 148 versus > 148, values which were selected for their correlation as they are the threshold for severe ARDS oxygenation in newly published criteria.^33^

A two-sided p value < 0.05 was considered statistically significant. Statistical analyses were conducted using Stata version 18.0 (StataCorp, College Station, TX). Reporting follows the Strengthening the Reporting of Observational Studies in Epidemiology (STROBE) statement (**Table E1**). This study was approved by the Johns Hopkins Medicine Institutional Review Board (IRB00513735) with a waiver of informed consent for secondary analysis of de-identified data.

## RESULTS

**Figure 1:**
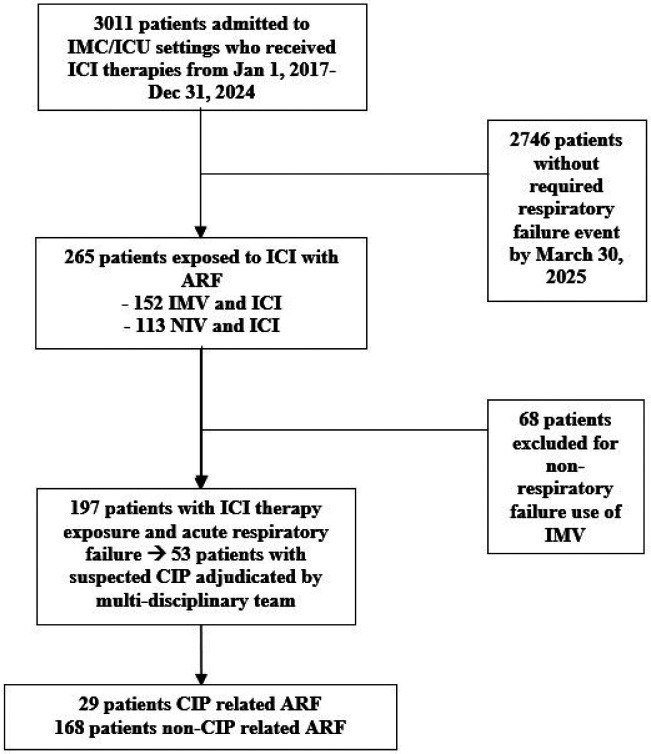
Flow Diagram of Patient Cohort Selection Flow diagram showing selection of ICI-treated patients with acute respiratory failure requiring ≥24 hours of invasive or noninvasive ventilatory support. Of 3,011 ICU or intermediate-care admissions, 197 patients met ARF criteria and comprised the analytic cohort, including 29 cases of CIP-related ARF and 168 non-CIP cases.

**Figure 2:**
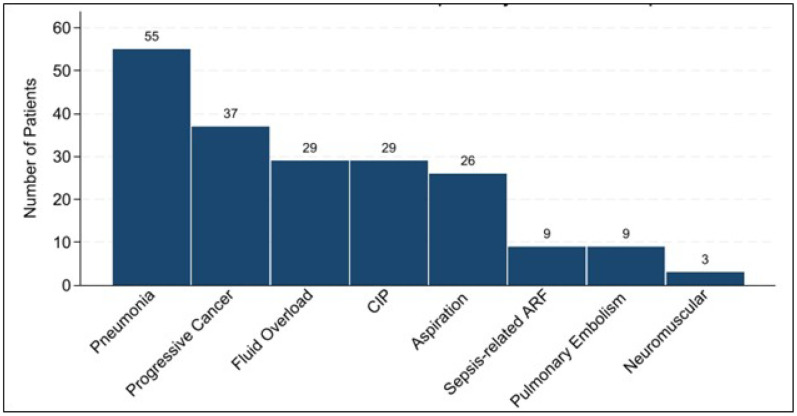
Distribution of Acute Respiratory Failure Etiologies Bar chart showing the number of patients by adjudicated cause of acute respiratory failure among immune checkpoint inhibitor–treated patients.

Among 3,011 patients in the registry who received an ICI in the JHHS, 197 (7%) met our definition of ARF requiring at least 24 hours of noninvasive or invasive ventilatory support for confirmed respiratory failure (Figure 1). Fifty-three patients were treated for suspected CIP at the bedside and underwent multidisciplinary adjudication. Among patients treated for suspected CIP by their inpatient clinicians, 55% (29/53) were ultimately adjudicated as having CIP-related respiratory failure. Over the entire cohort, this yielded 15% (29/197) with CIP-related ARF, and 85% (168/197) with non-CIP ARF. Agreement between pulmonology consensus adjudication and oncology review was perfect (κ = 1.00), with similarly high agreement between pulmonology and radiology reviewers (κ = 0.91). Among those with non-CIP ARF, the etiologies were pneumonia (n=54), progressive cancer (n=37), fluid overload (n=29), aspiration (n=26), sepsis-related respiratory failure (n=9), pulmonary embolism (n=9), and neuromuscular causes (n=3) (Figure 2). The distribution of primary cancer sites for the cohort is shown in **Figure E2.**

The median patient age was 66 years (interquartile range [IQR]: 59-74). Aside from lung cancer being more frequent in patients with CIP versus non-CIP (62 vs. 35% of patients), other baseline variables were similar between groups (Table 1). Patients in both groups had received a median of 3 ICI administrations prior to respiratory failure, and the distribution of initial ICI agents and classes, and total duration of ICI exposure were similar. The median time from first ICI exposure to ARF was 112 days in CIP (IQR: 56-209) and 138 days in non-CIP etiologies (IQR:54-332; p=0.44). CIP patients were more frequently managed with noninvasive ventilation alone (69% vs 45%), but more often experienced escalation from noninvasive to invasive ventilation compared with non-CIP patients (28% vs 11%; p<0.01). Although CIP patients had lower non-respiratory SOFA scores (median 2 vs 5; p<0.01), they presented with more severe hypoxemia, reflected by a lower nadir SpO_2_/FiO_2_ ratio in the first 24 hours (median of 120 vs 148; p=0.01). All CIP patients received systemic glucocorticoids compared with 68% of non-CIP patients (p<0.01), and among patients who received glucocorticoids, high-dose therapy (≥1 mg/kg methylprednisolone equivalent) was more common in CIP vs. non-CIP (90% vs 67%; p=0.02). Intravenous immunoglobulin was administered to 14% of CIP patients and 2% of non-CIP patients (p<0.01); use of other immunosuppressive agents was rare.

**Figure 3:**
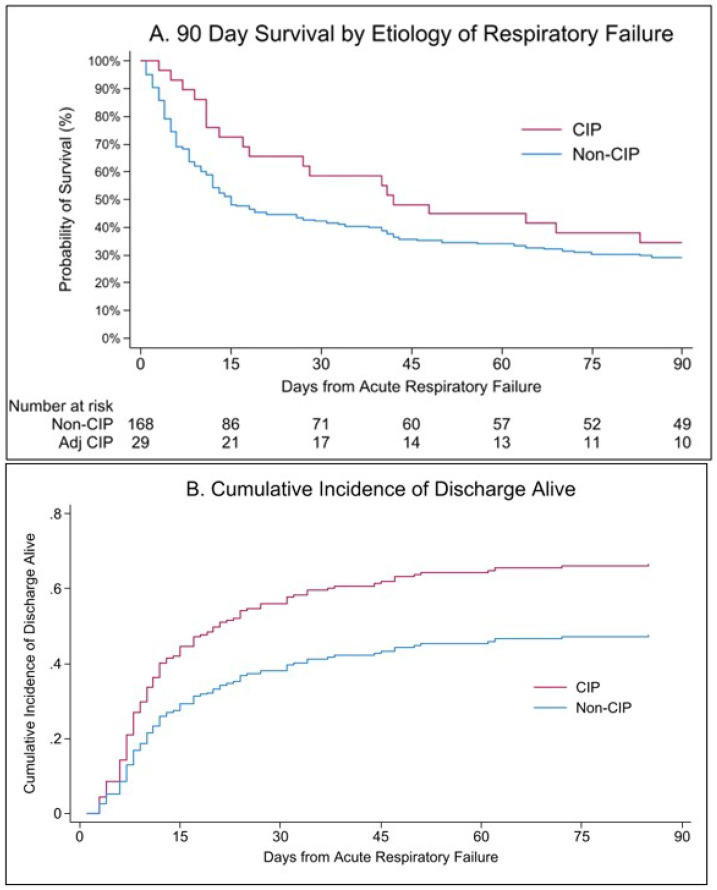
Ninety-Day Outcomes After Acute Respiratory Failure by Checkpoint Inhibitor Pneumonitis (CIP) Status (A) Kaplan–Meier estimates of 90-day survival following acute respiratory failure among patients with adjudicated CIP versus non-CIP etiologies. Numbers at risk are displayed below the x-axis. (B) Cumulative incidence of discharge alive over 90 days, accounting for the competing risk of in-hospital death, stratified by CIP status.

All-cause mortality by day 90 occurred in 65% of patients with CIP-related ARF and 71% of patients with non-CIP ARF (Table 2, Figure 3). In multivariable Cox proportional hazards models, time to death within 90 days did not differ significantly between CIP and non-CIP ARF (adjusted HR [aHR] 0.66; 95% CI, 0.40-1.08). In-hospital mortality was 31% in patients with CIP and 49% in patients with non-CIP ARF and was associated with a lower hazard of in-hospital death compared with non-CIP ARF (aHR 0.43; 95% CI, 0.21-0.88). Accounting for the competing risk of in-hospital death, patients with CIP-related ARF had a shorter time to discharge alive compared to non-CIP (sHR:1.70; 95% CI, 1.02-2.84) (Figure 3).

In patients who survived ARF hospitalization (n=106), a total of 14 patients (13.2%) restarted ICI therapy after recovery from ARF. Re-initiation of ICI occurred in 5.0% of adjudicated CIP cases who survived, and 16.9% of adjudicated non-CIP patients who were not treated for CIP (p=0.28). In patients in whom clinicians suspected and treated for CIP, but the multidisciplinary panel did not think was CIP, only 6.7% of survivors reinitiated ICI therapy. Among patients who restarted ICI therapy, the median time from recovery to rechallenge was 435 days.

Sensitivity analyses adjusting for recent cancer-directed therapies, including concurrent chemotherapy, definitive thoracic radiation, recent tyrosine kinase inhibitor exposure, and total duration of ICI therapy prior to ARF, also resulted in a non-significant difference in 90-day survival for CIP versus non-CIP, but did suggest the possibility of treatment-related confounding with a point estimate favoring non-CIP 1.32 (95% CI, 0.75-2.31). In analyses restricted to patients with non-small cell lung cancer (n=67), the adjusted HR for 90-day mortality was 0.74 (95% CI, 0.31-1.74) (Table 2**, Figure E2)**. When stratified by hypoxemia severity (SpO_2_/FiO_2_ ≤148 [n=96] vs >148 [n=82]), adjusted hazard ratios for 90-day mortality were 0.56 (95% CI, 0.30-1.03) and 1.34 (95% CI, 0.49-3.61) (Table 2, **Figure E3)**.

## CONCLUSION

In this retrospective cohort of patients who developed ARF within two years of initiating ICI therapy, CIP accounted for a minority of ARF cases. Ninety-day survival did not differ significantly between CIP and non-CIP ARF; however, patients with CIP experienced lower in-hospital mortality and shorter hospital length of stay. ICI therapy was infrequently reinitiated among survivors. Overall, the high morbidity and mortality observed in this cohort underscore the severity of acute respiratory failure in patients receiving immunotherapy.

Prior studies of CIP resulting in ARF, largely limited to NSCLC populations, have reported substantial in-hospital mortality and poor overall survival. In a recent cohort of patients with severe CIP, median hospital length of stay was 8 days, in-hospital mortality was 32%, and median overall survival was 4.4 months.^34^ Another study similarly reported a 29% mortality rate among patients with grade 3-4 pneumonitis, with deaths occurring exclusively in severe cases.^35^ In another real-world cohort, severe-grade CIP was associated with a median overall survival of 3.0 months and a CIP-related mortality rate of 22.7%.^36^ The in-hospital mortality observed in our CIP-related ARF cohort (31%) is comparable to these prior reports. Importantly, however, our study included patients across cancer types and additionally evaluated 90-day mortality, demonstrating that nearly two-thirds of patients with CIP-related ARF died within 90 days, suggesting substantial intermediate-term mortality beyond the index hospitalization.

Although CIP-related ARF was associated with lower in-hospital mortality, this short-term advantage did not translate into improved intermediate-term outcomes. This may reflect the potentially reversible nature of immune-mediated lung injury compared with other causes of ARF in this population, such as progressive malignancy or aspiration. However, outcomes following ICU-level ARF in oncology patients likely reflect not only the mechanism of lung injury but also the broader burden of malignancy and critical illness. ^4 37^ Notably, ICI therapy was infrequently reinitiated following ARF, even among patients ultimately adjudicated as not having CIP. The occurrence of ARF itself—independent of confirmed immune-mediated pneumonitis—appears to function as a clinical inflection point, frequently leading to permanent discontinuation of immunotherapy.

Despite access to multidisciplinary immunotherapy toxicity expertise within our health system, diagnostic uncertainty was common. Among patients treated for suspected CIP, just over half (55%) were ultimately adjudicated as having CIP-related respiratory failure. This reflects the significant overlap in presenting features between CIP and alternative etiologies such as infection, tumor progression, and aspiration.^37, 38^Although multidisciplinary adjudication is increasingly recommended as a gold standard approach for CIP diagnosis, real-time access to such expertise remains limited in many settings.^17, 25-27^ Structured diagnostic pathways incorporating radiology input, targeted microbiologic evaluation, and timely multidisciplinary review may help reduce misclassification, limit unnecessary immunosuppression, and preserve opportunities for ICI continuation when appropriate.

This study has several limitations. The number of adjudicated CIP cases was modest, limiting statistical power and precision. As an observational study, residual confounding is possible despite multivariable adjustment, and unmeasured differences in underlying cancer burden, functional status, or frailty could have influenced both ARF etiology and mortality risk. Misclassification of CIP diagnosis remains possible despite multidisciplinary adjudication, reflecting the inherent diagnostic uncertainty of CIP in critically ill patients, and may have influenced observed associations between ARF etiology and outcomes. Finally, the single health-system design may limit generalizability to settings with different patient populations, ICU admission practices, or access to multidisciplinary immunotherapy toxicity expertise.

In summary, ARF after ICI therapy arises from heterogeneous etiologies, with CIP accounting for a minority of cases. CIP-related ARF was not associated with differential 90-day survival but was associated with lower in-hospital mortality and lower subsequent ICI reinitiation. These findings underscore the clinical complexity of ARF in ICI-treated patients and highlight the importance of precise etiologic attribution to inform subsequent immunotherapy decision-making.

## Supplementary Material

This is a list of supplementary files associated with this preprint. Click to download.

• SupplementalMaterials.docx

## Figures and Tables

**Table 1:** Clinical Characteristics of Acute Respiratory Failure Cases after Immune Checkpoint Inhibitor Therapy, Stratified by Etiology of Acute Respiratory Failure

	Total (n= 197)	CIP- related ARF (n=29)	Non-CIP ARF (n=168)	*P-* *value*
**Baseline Characteristics**				
Age at admission, years	66 (59-74)	70 (64-76)	65 (58-74)	0.11
Female	67 (34%)	8 (28%)	59 (35%)	0.43
Race				0.82
White	125 (64%)	17 (59%)	108 (64%)	
Black	55 (28%)	10 (35%)	45 (27%)	
Asian	12 (6%)	1 (3%)	11 (7%)	
Other	5 (2%)	1 (3%)	4 (2%)	
Hispanic Ethnicity	17 (9%)	1 (3%)	16 (10%)	0.28
Body mass index	25 (22-30)	25 (22-30)	25 (22-29)	0.90
Ever smoker	126 (64%)	21 (73%)	105 (63%)	0.30
Elixhauser comorbidity count	3 (1-5)	3 (2-5)	3 (1-5)	0.13
Lung Cancer	77 (39%)	18 (62%)	59 (35%)	0.01
Cancer Stage				0.91
I	4 (2%)	1 (3%)	3 (2%)	
II	11 (6%)	2 (7%)	9 (5%)	
III	33 (17%)	5 (17%)	28 (17%)	
IV	140 (71%)	19 (66%)	121 (72%)	
Incomplete/Unknown	9 (4%)	2 (7%)	7 (4%)	
Brain Mets	54 (27%)	7 (24%)	47 (28%)	0.67
**Immune Checkpoint Inhibitor Therapy Characteristics**				
Number of ICI Administrations Prior to Respiratory Failure	3 (2-8)	3 (2-5)	3 (2-9)	0.54
First ICI Administered				0.47
Atezolizumab	15 (8%)	3 (10%)	12 (7%)	
Cemiplimab	8 (4%)	0	8 (5%)	
Dostarlimab	1 (0.5%)	0	1 (0.5%)	
Durvalumab	15 (8%)	5 (17%)	10 (6%)	
Ipilimumab-Nivolumab	20 (10%)	4 (14%)	16 (10%)	
Nivolumab	34 (17%)	5 (17%)	29 (17%)	
Nivolumab-Relatlimab	2 (1%)	0	2 (1%)	
Pembrolizumab	101 (51%)	12 (42%)	89 (53%)	
Tremelimumab-Durvalumab	1 (0.5%)	0	1 (0.5%)	
Total duration of ICI exposure (time from first ICI receipt to last ICI receipt occurring before respiratory failure) (days)	52 (21-156)	43 (21-84)	54 (21-182)	0.27
Median time from first ICI to Respiratory Failure (days)	133 (54-316)	112 (56-209)	138 (54-332)	0.44
**Other Cancer Therapies**				
Chemotherapy concurrent with ICI	68 (35%)	11 (38%)	57 (34%)	0.68
Thoracic radiation therapy				
Palliative dose	28 (14%)	6 (21%)	22 (13%)	0.28
Definitive dose	24 (12%)	9 (31%)	15 (9%)	<0.01
Prior TKI therapy	5 (3%)	1 (3%)	4 (2%)	0.74
**Acute Respiratory Failure Event Characteristics**				
Admitted to academic hospital	147 (75%)	21 (72%)	126 (75%)	0.77
Respiratory support				
Non-invasive Ventilation Only (NIV)	96 (49%)	20 (69%)	76 (45%)	<0.01
Invasive Ventilation Only (IMV)	75 (38%)	1 (3%)	74 (44%)	
Both NIV and IMV	26 (13%)	8 (28%)	18 (11%)	
Respiratory virus positive	26 (13%)	3 (10%)	23 (14%)	0.62
Coronavirus 19 positive	13 (7%)	1 (3%)	12 (7%)	0.33
Lowest SpO2/FiO2 ratio in first 24 hours	136 (115-168)	120 (110-139)	148 (116-182)	0.01
Non-respiratory SOFA score	5 (2-7)	2 (1-5)	5 (2-8)	<0.01
Vasopressor use	62 (32%)	4 (14%)	58 (35%)	0.03
Other iRAE (any organ)	13 (7%)	0	13 (8%)	0.12
**Other Therapies Received During Hospitalization**				
Intravenous antibiotics for ≥ 48 hours	129 (66%)	18 (62%)	111 (66%)	0.68
Any corticosteroid	141 (72%)	29 (100%)	114 (68%)	<0.01
≥ 1 mg/kg methylprednisolone equivalent	140 (71%)	26 (90%)	112 (67%)	0.02
Intravenous Immunoglobulin	7 (4%)	4 (14%)	3 (2%)	<0.01
Infliximab	0	0	0	
Tocilizumab	0	0	0	
Mycophenolate mofetil	3 (2%)	0	3 (2%)	0.08

Data are shown as median (interquartile range) for continuous variables and n (%) for categorical variables.

Abbreviations: ARF, acute respiratory failure; CIP, checkpoint inhibitor pneumonitis; ICI, immune checkpoint inhibitor; NIV, non-invasive ventilation; IMV, invasive mechanical ventilation; SOFA, Sequential Organ Failure Assessment; SpO_2_/FiO_2_, peripheral oxygen saturation to fraction of inspired oxygen ratio; iRAE, immune-related adverse event; TKI, tyrosine kinase inhibitor; COVID-19, coronavirus disease 2019.

**Table 2:** Association Between CIP-Related Respiratory Failure and Outcomes

Outcome	CIP-related ARF	Non-CIP- related ARF	Unadjusted Analysis HR (95% CI)	Multivariable Analysis* HR (95% CI)
**Primary Outcome**				
90-day all-cause mortality (n=188)	19 (65%)	119 (71%)	0.73 (0.45-1.18)	0.66 (0.40-1.08)
**Secondary Outcomes**				
In-hospital mortality (n=188)	9 (31%)	82 (49%)	0.52 (0.26-1.04)	0.43 (0.21-0.88)
	Median Days [IQR]	Median Days [IQR]		
Time to hospital discharge (n=188)	11 (9-14)	12 (7-27)	--	SHR 1.70 (1.02-2.84)
**Sensitivity/Exploratory Analyses**				
Analysis Including Pre-ARF Additional Cancer Therapeutics (n=188)	--	--	--	1.32 (0.75-2.31)
NSCLC only (n=67)	15 (52%)	52 (31%)	0.59 (0.27-1.31)	0.74 (0.31-1.74)
S/F ≤148 (n=96)	22 (23%)	74 (77%)	0.65 (0.35-1.19)	0.58 (0.31-1.09)
S/F >148 (n=82)	7 (9%)	75 (91%)	0.98 (0.42-2.27)	1.29 (0.51-3.23)

* Adjusted for age, sex, Elixhauser comorbidity count, smoking status, presence of other immune-related adverse events, lung, and cancer stage.

Abbreviations: ARF, acute respiratory failure; CIP, checkpoint inhibitor pneumonitis; NSCLC, non-small cell lung cancer; SHR, sub-hazard ratio; S/F, SpO2/FiO2

## Data Availability

The datasets generated and/or analyzed during the current study are not publicly available due to institutional privacy regulations but are available from the corresponding author on reasonable request and with appropriate Institutional Review Board approval.
